# Infodemic in Germany and Brazil: How the AfD and Jair Bolsonaro are Sowing Distrust During the Corona Pandemic

**DOI:** 10.1007/s41244-021-00210-6

**Published:** 2021-08-09

**Authors:** Dinah K. Leschzyk

**Affiliations:** Institute for Romance Studies, Justus Liebig University Giessen, Gießen, Germany

**Keywords:** Crisis Communication, Corona Communication, Bolsonaro, AfD, Right-Wing Populism, Infodemic, Disinformation, Discreditation, Credibility, Krisenkommunikation, Corona-Kommunikation, Bolsonaro, AfD, Rechtspopulismus, Infodemie, Desinformation, Diskreditierung, Glaubwürdigkeit

## Abstract

Even though the topic of *infodemic* – a blending of the words *information* and *pandemic* – emerged just in 2020 it addresses a question that has been crucial ever since in communication: How to establish – or undermine – credibility? This article deals with rhetorical techniques applied by Brazilian President Jair Messias Bolsonaro (2019–) and high-ranking politicians of the German party AfD (*Alternative für Deutschland*) during the COVID-19-pandemic. The analysis is based on tweets published through their official accounts during the first year of the pandemic (*@jairbolsonaro, @AfD*). Meanwhile Bolsonaro, who was in charge during the crisis, attacks the media claiming they would spread panic and false information, AfD, an opposition party, concentrates its criticism on the federal and state governments. The key concept *credibility*‹ as discussed by Ortwin Renn (2019) and basic claims that appear in Aristotle’s »Rhetoric«, dating from the 4th century BC, build the theoretical basis of this study. Methodologically, the analysis is based on the *Discourse-Historical Approach* (Reisigl/Wodak 2001), focusing on discursive strategies of negative other representation, and a framework for studies on the language of legitimation and delegitimation developed by Theo van Leeuwen (1996).

## Abstract (de)

In der sogenannten *Infodemie* – eine Wortmischung aus *Information* und *Pandemie* –, die 2020 aufkam, ist eine Frage zentral, die seit jeher grundlegend für die Kommunikation ist: Wie lässt sich Glaubwürdigkeit erzeugen bzw. untergraben? Gegenstand des vorliegenden Artikels ist die Rhetorik des brasilianischen Präsidenten Jair Messias Bolsonaro (2019-) sowie hochrangiger Politiker der deutschen Partei AfD (*Alternative für Deutschland*) im Rahmen der COVID-19-Pandemie. Die Analyse basiert auf Tweets, die im ersten Jahr der Pandemie über die offiziellen Twitter-Accounts (*@jairbolsonaro, @AfD*) veröffentlicht wurden. Während Bolsonaro, der während der Krise amtierender Präsident ist, die Medien verbal attackiert und behauptet, diese würden Panik und Falschinformationen verbreiten, konzentriert sich die AfD, eine Oppositionspartei, mit ihrer Kritik auf die Regierungen von Bund und Ländern. Der Schlüsselbegriff *Glaubwürdigkeit*, definiert nach Ortwin Renn (2019), sowie Feststellungen, die bereits Aristoteles in seinem Werk *Rhetorik* (4. Jhd. v. Chr.) traf, bilden die theoretische Grundlage der Studie. Methodisch stützt sich die Analyse auf den *Diskurshistorischen Ansatz* (Reisigl/Wodak 2001), der u. a. auf diskursive Strategien der negativen Fremddarstellung abzielt, sowie ein von Theo van Leeuwen (1996) entwickeltes Schema zur Kategorisierung von Strategien der Legitimierung und Delegitimierung.

## Introduction

The corona pandemic in 2020/21 came along with a special kind of information crisis that had never been seen before, where claiming to tell ›the truth‹, accusing the others to lie, deceive and sow panic was common. Rupali Jayant Limaye et al. (2020) observe: »Unlike historical pandemics […], COVID-19 is spreading across a highly connected world, in which virtually all individuals are linked to each other through the mobile phone in their pockets.« For the phenomena in this context a new term has been created: *infodemic*. In a joined statement of WHO, UN, UNICEF, and other big international players on »Managing the COVID-19 infodemic: Promoting healthy behaviours and mitigating the harm from misinformation and disinformation« *infodemic* is defined as »an overabundance of information, both online and offline«, that »includes deliberate attempts to disseminate wrong information to undermine the public health response and advance alternative agendas of groups or individuals« (2020). As the international organizations outline:

Misinformation costs lives. Without the appropriate trust and correct information, diagnostic tests go unused, immunization campaigns […] will not meet their targets, and the virus will continue to thrive. [D]isinformation is polarizing public debate […]; amplifying hate speech; heightening the risk of conflict, violence and human rights violations; and threatening long-terms prospects for advancing democracy, human rights and social cohesion.

In an earlier statement the WHO (2020) links these misleading news, images, and videos with its wording directly to the pandemic, observing that »[l]ike the virus, it is highly contagious and grows exponentially.« The WHO Director-General at the time, Tedros Adhanom Ghebreyesus (2020), states: »We’re not just battling the virus, we’re also battling the trolls and conspiracy theorists that push misinformation and undermine the outbreak response.«

To this worldwide phenomena Germany and Brazil have been of no exception, even though the political and pandemic situation in both countries was – and is by now (June 2021) – completely different: Whereas in Brazil a far right populist president keeps downplaying the virus and its effects, the German government, led by long-term Chancellor Angela Merkel (*Christian Democratic Union of Germany*, CDU), took the virus and it’s risks absolutely serious right from the beginning, constantly implying measurements to »flatten the curve« of infections. It is a common place that times of crises are times of governments (see for example Decker/Ruhose 2021, p. 1) as being in charge offers the possibility to handle the situation and therefore being perceived as *the savior*. The opposition on the other hand can either support the governmental decisions or criticize them – both strategies tend to have positive and negative implications and possible consequences for the actors. In Germany there was one party that started to criticize harshly on the government’s actions almost right from the beginning of the pandemic, the so called *Alternative für Deutschland*, ›Alternative for Germany‹ (AfD). This article focuses on a crucial claim of these statements, that is: This government cannot be trusted. In the Brazilian case, on the other side, the president attacks the trustworthiness of the media.

The main actors, Jair Bolsonaro and the AfD, will be presented in the following section (2.) together with the political and pandemic situation in Germany and Brazil. Questions concerning the credibility of actors are among the oldest in rhetoric theory as will be shown in section 3. The key term *credibility* with its rather wide notion will be analyzed in the same section. The framework underlying the empirical analysis and the corpus will be described in section 4. The findings of the analysis will be presented in section 5. In the last paragraph (6.) a resume and final considerations will be given. All tweets published by Bolsonaro and the AfD that are cited in this article are listed in the tables 4 and 5 in the appendix.

## The political and pandemic context in Brazil and Germany

As early as February 2020 Frank Sieren, journalist of the *Deutsche Welle*, comments on the extraordinary speed with which misinformation and conspiracy theories^1^ are spreading in the coronavirus crisis (cf. Sieren 2020). This is especially true for Brazil, where the fact checking services had a tough time during the pandemic. *Aos Fatos* for example checked the statements of Brazilian President Jair Bolsonaro on COVID-19 and found that during a period of six month (March to September 2020) more than 600 of his statements on the pandemic were false or distorted (cf. Ribeiro/Cunha 2020).

Although Bolsonaro spent 30 years in politics, he positions himself as anti-establishment, hence following a fundamental populist principle (cf. Müller 2017, p. 26). In 1990 he was elected federal deputy for Rio de Janeiro and has not left the Chamber of Deputies until 2018, the year he was running for president (cf. Câmara dos Deputados 2019). He was elected in the second-round runoff and has been in the office since the 1^st^ of January 2019. Due to a limited airtime on television – amongst other reasons – he and his team have been using social media excessively during the presidential campaign. Vanice Maria Oliveira Sargentini and Geovana Chiari (2019, p. 459) observe: »Com pouco tempo de propaganda na televisão, Bolsonaro, a exemplo de D. Trump, aposta nos Tweets, em publicações de mensagens no Facebook e WhatsApp, centralizando, assim, seu canal de comunicação com os eleitores nas mídias digitais.«^2^ The nickname »Tropical Trump«, that media adopted straight from the beginning of his presidency (see for example the headlines of Xing (2019) and the *Global Times* (Editorial Board 2020)), seems very well earned, when comparing not only his social media use but also his statements on the media, women and minorities to the ones of former US-president Donald Trump. Due to his statements, actions and communication strategies Bolsonaro can be positioned as far right, fascist and populist. He disrespects the institutions, especially the Supreme Court (Pt. *Supremo Tribunal Federal*, STF), is pro-arms, anti-liberalization of abortion laws and against same-sex-marriage. Bolsonaro rejects the scientific consensus on climate change and is downplaying the corona pandemic (cf. Leschzyk 2020 pp. 107 f.) in which, so far (June 2021), more than 501.825 people have died in Brazil (cf. JHU 2021).

In terms of the pandemic the situation in Germany is far less dramatic than it is in Brazil. By now, June 2021, 90.400 people who have been diagnosed with an infection have died (cf. JHU 2021). The health care system is not about to collapse, so far everybody in need can be hospitalized. Anyways, the governments measurements to contain the pandemic have been criticized by the AfD continuously. The AfD was founded in 2013, a year that has been diagnosed a »[*p*]erfect starting point for populists […]« (Amann 2017, p. 17, translation D.L.) because of its overall climate of crisis. With reference to Frank Decker (2004) Fedor Ruhose (2020, p. 16) considers »political opportunity structures« (translation D.L.) a necessity for the successful creation of a populist party. The AfD was called a »Professor’s Party« as some of its most prominent figures had been working at universities at some point in their life, including co-founder Bernd Lucke. Besides this curiosum, the party was highly mixed and ideologically without contours. The members were related by a »common rejection of the world« and a »negative community spirit« as Melanie Amann (2017, p. 56, translation D.L.) states. In 2015 the party had a »turn to the right«, that led to a right wing/far right party with a right-wing extremist part that is being monitored by the constitution protection service (Ger. *Verfassungsschutz*). The AfD was extremely successful in 2016, where »[t]he broad consensus among the parties represented in parliament […] had created a vacuum« (Köcher 2016, p. 7, translation D.L.). Nowadays the party positions itself as anti-immigration, anti-European integration, pro ›traditional‹ gender roles and against same-sex marriage (cf. Alternative für Deutschland 2021). They criticize the established political parties, the media, and what they call ›the elites‹ in general. In election polls the voting intentions circulate between 9 and 15 percent (cf. IfD Allensbach 2021).

## The theoretical background

When looking at the corona infodemic through a theoretical lens, a broad field opens. There are typical elements of populism, like criticism of the elites and the media; the claim to speak for ›the people‹ as a homogeneous mass with one will, that only populists get to know; and a strong polarization in *we* vs. *them* that works with stigmatization, fear mongering and often hate speech (for a characterization of populism see for example Amann 2017; Jörke/Selk 2017; Mudde/Rovira Kaltwasser 2017; Müller 2017; Wodak 2015). As soon as the corona pandemic started, several studies on the communication during the crisis have been published. One to point out is *More Than Words: Leaders’ Speech and Risky Behavior during a Pandemic* (2020) by Nicolás Ajzenman, Tiago Cavalcanti and Daniel Da Mata. The researchers’ key finding is that Bolsonaro’s supporters follow experts’ advice less than his non-voters. They conclude:

We find that after Brazil’s president publicly and emphatically dismisses the risks associated with the COVID-19 Pandemic and advises against isolation, social distancing measures of citizens in pro-government localities reduce relative to those places in which his support is weaker […]. The impact is large and robust to different empirical model specifications (Ajzenman et al. 2020).

As mentioned before, two of the key words for corona infodemic are *disinformation* and *misinformation*; while *disinformation* can be defined as »knowingly false content meant to deceive« (Brennen et al. 2020, p. 2), something that is difficult to prove, *misinformation* is used to »refer broadly to any type of false information – including disinformation« (ibid.).^3^ The WHO et al.(2020) considers mis- and disinformation as possibly »harmful to people’s physical and mental health«, due to the fact that it can »increase stigmatization; […] and lead to poor observance of public health measures, thus reducing their effectiveness and endangering countries’ ability to stop the pandemic.«

A salient feature of this infodemic is, that actors claim to tell ›the truth‹, to refer to ›the facts‹, while others would spread false information. If it is not possible to determine from one’s own experience whether claims are correct, the question is, whose information is trustworthy. Therefore, *credibility* is a key concept (cf. Renn 2019, p. 77). The concept has a rather wide notion. It can be narrowed down to basic definitions like: *Credibility* is »the fact that someone can be believed or trusted« (Cambridge Dictionary 2014); »[i]f someone or something has credibility, people believe in them and trust them« (Collins Dictionary n.d.); *credibility* is »the quality of deserving to be believed and trusted« (Longman Dictionary 2020). For Ortwin Renn (2019, pp. 77 f., translation D.L.), a person or institution is said to be credible »if the information we receive from them is true (in accordance with the facts) and truthful (not distorting its own intention) to the best of the sender’s knowledge and belief«.^4^ Renn (2019, p. 77, translation D.L.), sociologist specialized in risk research, refers to credibility and trust as »sensitive resources«. Marlene Odenbach (2005, p. 194, translation D.L.) speaks of *credibility* in a similar way, describing it as a »highly sensitive, fragile asset« that, once damaged, only can be restored at great expense (time and money). And – if it can be restored at all – »glued [...] is just not as good as new« (ibid.).

*Credibility* is – together with *reliability* that is referred to as *performance* (cf. Williams 1985 as cited in Renn 2019, p. 78, translation D.L.) – a central component of trust. *Trust* means »granting a person or institution [...] that they are doing the right and sensible thing in each case, even if one cannot comprehend or verify the motives in detail« (Renn 2019, p. 78, translation D.L.). *Performance* in that sense means »that the respective institution properly fulfills its task in the sense of the given mission [...]« (Renn 2019, p. 78, translation D.L.). Both components can further be split up: »credibility […] is tied to openness, transparency, and honesty; performance to competence, fairness, and commitment« (Renn 2019, p. 78 in reference to Renn/Levine 1991, pp. 179 f., translation D.L.). Another key criterion for attributing trust is *straightforwardness*, which is tied to both components – *credibility* and *performance* (ibid.). These criteria for attributing trustworthiness are listed in the following table 1.

**Table 1:** Criteria for the attribution of trustworthiness (according to Renn 2019, pp. 78 f., translation D.L.)

**Components of trust**	**Attributed criteria**
credibility	openness transparency honesty
	straightforwardness
performance	
	competence fairness commitment

The fundamental loss of trust in the credibility of sources is accompanied by insecurity and cynicism. Renn (2019, p. 84, translation D.L.) speaks of »insecure to cynical people who have lost basic trust in groups and institutions of knowledge transfer and have only derision and contempt left for the seemingly public-spirited institutions.« Populists, for their part, »deliberately tap into the insecurity caused by a lack of loyalty to conventional institutions such as science, politics, or authorities, promising security and safety if one would only confide in them« (Renn 2019, p. 84, citing Milbradt 2018, p. 206, translation D.L.). Keeping that in mind, one of the oldest questions of rhetorical studies emerges: How do actors establish their trustworthiness while undermining that of their opponents? These questions have already been addressed by Aristotle. In his magnum opus *Rhetoric* (II, 1378a [2015, p. 67]) dating from the 4th century BC, Aristotle comments on the question how to appear a credible person: »There are three things which inspire confidence in the orator’s own characterhe three, namely, that induce us to believe a thing apart from any proof of it: good sense, good moral character, and goodwill.« There are obviously significant overlaps to the criteria of trustworthiness identified by Renn who uses the modern expressions *competence, straightforwardness, fairness* and so on (see table 1). As »the objects of praise and blame« Aristotle (I, 1366a [2015, p. 35]) considers »Virtue and Vice« and »the Noble and the Base«. When the philosopher talks about »forms of Virtue« (I, 1366a [2015, p. 36]) he refers to »justice, courage, temperance, magnificence, magnanimity, liberality, gentleness, prudence, wisdom«. As effective rhetorical ways of blaming or praising a person Aristotle (I, 1368a [2015, pp. 40 f.]) then mentions »heightening the effect« – as to say *intensification *–, *comparison*, and *examples*. These techniques will be kept into mind, when analyzing the communication of populist actors during the corona crisis. In the following section the data material of the analysis and the methodological approach will be introduced.

## The analytical framework and the corpus

Beyond the classical Aristotelean approach presented in section 3. the analytical framework for the analysis of techniques for establishing – or undermining – trustworthiness involves discursive strategies of positive self- and negative other-presentation. This type of strategy – strategy is understood as »a more or less accurate and more or less intentional plan of practices (including discursive practices) adopted to achieve a particular social, political, psychological or linguistic aim« (Reisigl/Wodak 2001, p. 44) – is addressed by the *Discourse-Historical Approach* (DHA) as presented in »Discourse and discrimination. Rhetorics of Racism and Antisemitism« (2001) by Martin Reisigl and Ruth Wodak. As Reisigl (2017, p. 92) points out, there are several linguistic techniques that can be used in the context of predicational strategies – an observation that also applies to the other strategies. Reisigl/Wodak (2001, pp. 44 f.) list key questions that can be helpful to identify these different strategies. As this analysis is focused on negative other-presentation, which is considered predominant in the discourse, meanwhile self-presentation plays a minor role, the strategies of nomination and predication are given special attention (see table 2).

**Table 2:** Categories and key questions of the analysis (cf. Reisigl/Wodak 2001, pp. 44 f.; Renn 2019, pp. 78 f.; van Leeuwen 2013, pp. 327 f.)

**Referential/Nomination strategies**	How are actors named and referred to regarding the components of trust?
**Predicational strategies**	What characteristics are attributed to actors when it comes to their credibility and
**(De-)legitimation strategies**	Which categories (authorization, moral evaluation, rationalization, mythopoesis) are addressed to (de-)legitimate claims?

The DHA is compatible with a framework for analyzing the language of legitimation and delegitimation by Theo van Leeuwen (1996)^5^, strategies that also come into play when undermining credibility. Van Leeuwen (2013, p. 330) characterizes legitimation »as an answer to the spoken or unspoken ›why‹ question – ›Why should we do this?‹ or ›Why should we do this in this way?‹« The analysis of these strategies concerns the question of what is referred to for making statements seem plausible (strategies of legitimation) or implausible (strategies of delegitimation). The focus here lies on strategies of delegitimation, as to say: By reference to what or whom do actors invalidate the statements of a) the government (AfD) and b) the media (Bolsonaro)?

Van Leeuwen (2013, pp. 327 f.) identifies four major categories of legitimation: *authorization, moral evaluation, rationalization* and *mythopoesis* (see table 2). As he shows, »one of the ›forms and contents of legitimation‹ is, ›because I say so‹, where the ›I‹ is someone in whom some kind of authority is vested, or ›because so-and-so says so‹, where the authority is vested in ›so-and-so‹« (van Leeuwen 2013, p. 330). *Authority* can be categorized into *Personal Authority, Expert Authority, Role Model Authority, Impersonal Authority, The Authority of Tradition* and *The Authority of Conformity* (cf. van Leeuwen 2013, pp. 330–334). *Moral legitimation* can further be differentiated into *Evaluation, Abstraction* and either positive or negative *Comparison* (cf. van Leeuwen 2013, pp. 334–337). As van Leeuwen (2013, p. 338) observes, »[i]*n* the case of moral evaluation, rationality has gone underground.« Meanwhile »in the case of rationalization, morality remains oblique and submerged, even though no rationalization can function as legitimation without it« (ibid.). (De‑)Legitimation by rationalization can be distinguished in *Instrumental Rationalization* and *Theoretical Rationalization* (cf. van Leeuwen 2013, pp. 338–343): »Instrumental rationality legitimates practices by reference to their goals, uses and effects. Theoretical rationality legitimates practices by reference to a natural order of things […]« (van Leeuwen 2013, p. 338). The fourth category of (de-)legitimation concerns storytelling as in moral tales and cautionary tales (cf. van Leeuwen 2013, p. 343).

As pointed out before, Bolsonaro is being called »Tropical Trump« and like his US-role model he uses social media excessively. This calls out for the analysis of texts that have been published through this type of media. For this study I have built a corpus of tweets that have been published on his official account *@jairbolsonaro* throughout the first year of the pandemic (2020). The first time that the word *corona* has been used by Bolsonaro in a tweet was the 31^st^ of January 2020. That sets the starting date for the data collection that ended the 28^th^ of February 2021. In total *corona* appears 79 times in Bolsonaro’s tweets and retweets, meanwhile the term *covid* is used 193 times.

It is interesting that in the German context it is the AfD that is dominating the field of social media in comparison to the other political parties. On *@AfD*, the party’s official account on Twitter, the first tweet on the corona pandemic is published almost the same day as on Bolsonaro’s account: the 1^st^ of February 2020. Even the total number of (re-)tweets on the two accounts is similar with 2727 published through *@AfD* and 2859 on the channel *@jairbolsonaro*. The same applies to the number of (re-)tweets that belong directly to the corona discourse: 576 (*@AfD*) and 561 (*@jairbolsonaro*). Unlike Bolsonaro the AfD uses the term *corona* more frequently than the word *covid* (see table 3 for these stats). Besides similarities in quantity, it is to be expected that there will be used similar statements and arguments by both political actors. As there are strong ties between far-right positions all around the world the same discursive strategies, rhetorical techniques, and keywords are used in settings that are as different as Brazil and Germany.

**Table 3:** The data material (31.01.2020–28.02.2021)

**Username**	**number** **(re-)tweets**	**corona discourse**	corona*	covid*
*@AfD*	2727	576	346	56
*@jairbolsonaro*	2859	561	79	193

The analysis software MAXQDA Analytics Pro (version 2018.2) was used to process the data material. It is a paid software of the company VERBI, by means of which qualitative and quantitative data analyses can be supported. It enables automated searches and allows, for example, to determine the quantities of single terms and collocations.

## Undermining trustworthiness in the corona pandemic 2020/21

This section addresses the questions of how the AfD affronts the government’s trustworthiness and how Bolsonaro attacks the trustworthiness of the media during the COVID-19-pandemic. The concept of *trustworthiness* is operationalized by using Renn’s (2019, p. 87 f.) criteria bearing in mind that already Aristotle mentions *good sense* as to say *expertise, character* and *goodwill* as fundamental aspects of how to appear a credible person (see section 3.). All posts excerpted in this paper are listed in the appendix with their respective URLs (tables 4 and 5).

### AfD’s attacks on the trustworthiness of the government

In the AfD’s corona discourse on Twitter (576 tweets and retweets on *@AfD*) the German Chancellor Angela Merkel is addressed 66 times directly by her name, of which 41 times a hashtag is being set (*#Merkel*). Her first name is used four times and there are four references to her political office (*Bundeskanzlerin, Kanzlerin*). Merkel is attributed as ›arrogant‹, ›ideologically stubborn‹, acting without considering the facts (›fact-free‹) and – in general – lacking leadership (›not leading, only reacting‹).^6^ These are predications that have a negative impact on the performance of any politician and therefore are suitable to diminish the trustworthiness of the Chancellor. The drastic demand *Merkel must go* is used twice (tweets 24, 37). In a retweet from Stephan Brandner (*@StBrandner*), member of the *Bundestag* (German federal parliament), whose radical rhetoric in the corona discourse is particularly striking, Merkel is referred to as *millionenfache Verfassungsbrecherin* (Eng. ›million times Constitution breaker‹)^7^, who should be ›locked up‹ together with ›her accomplices‹ – a term (Ger. *Helfershelfer*) that is often used in the context of the Holocaust:

(1) RT @StBrandner: Meine Rede: Die **millionenfache Verfassungsbrecherin #Merkel und ihre Helfershelfer einsperren**! (tweet 26).

The topos of illegitimacy is addressed frequently in AfD’s corona discourse when it comes to discredit the government. The (de-)legitimation strategy therefore is *reference by authorization*: The Constitution says so and so (type: ›impersonal authority‹). That the government’s crisis management would not match democratic principles at all is expressed by the term *Diktatur* (Eng. ›dictatorship‹) (see tweet 28), that is used in the rhetoric of AfD’s politicians frequently (ten times in the overall data material, three more times as adjective: *diktatorisch*). This reference follows the *strategy of moral delegitimation* by a negative comparison of the government’s crisis management to a form of government characterized by no toleration for freedom of expression, equality, consent, voting and other democratic values. The same strategy applies when Merkel is being accused of having an ›anti-democratic attitude‹ (tweet 40). Besides the claim of a *#Corona-Diktatur* the AfD creates the term *#Coronakratur* based on the Greek word *kratos* (*κράτος*) for ›state‹ to evoke a system of government where *corona* is the power source and in which ›all prime ministers dance to Merkel’s tune‹ – an attack on the prime ministers’ ›straightforwardness‹ – a central component of trustworthiness:

(2) RT @StBrandner: #5Fragen5Antworten […]: **#Coronakratur** bis in alle #Ewigkeit? Wird es jemals ein #Lockdownende geben? Wieso **tanzen alle #Ministerpräsidenten nach #Merkel|s Pfeife?** (tweet 1).

Suggestive questions as seen in example 2 are used on a regular basis in tweets published through *@AfD*. This rhetorical technique insinuates that the sender is stating the obvious, meanwhile an allegation – that is not questioned – is made.

Furthermore, the *topos of illegitimacy* is addressed by Alice Weidel, leader of the AfD in the Bundestag, who claims that ›Merkel turns violation of the law into a political principle‹ (tweet 32). She combines this allegation in the following tweet with an attack on the overall *competence* of the grand coalition (*Große Koalition*, often abbreviated as *GroKo*)^8^ by the word *Totalversagen* (›total failure‹):

(3) RT @Alice_Weidel: **Merkel macht Rechtsbruch zum politischen Prinzip**! Die **#GroKo** glänzt durch **Totalversagen**. Von Wirtschaft über Klima und Justiz bis hin zu Flüchtlingspolitik und **#Corona-Wahn** – alles haben #Merkel und ihre Regierung **in den Sand gesetzt**. Meine **Generalabrechnung** seht Ihr im Video: https://t.co/CKTKcvgd4W (tweet 32)

The supposed ›failure‹ is part of a fundamental critique of the government’s ›performance‹ during the corona crisis and another topos that is frequently referred to by the AfD. The term *versagen* (Eng. ›failure‹) is used six times in the tweets and retweets, one more time with reference to the federal government (*#Regierungsversagen*), two times referring explicitly to Merkel and the topic *vaccinations* (*#Merkel ihr #Impf-Versagen*; *sie wegen des Impf-Versagens*), another time implicitly to the government and other parties that are called *#Altparteien*^9^ in the rhetoric of AfD’s politicians (*#Politik-Versagen*) and one time to the government of the state Thuringia (*Versagen der Landesregierung*).^10^

The attribution ›crazy‹ evoked by the word *Wahn* as in *#Corona-Wahn* (see example 3), downgrades the government’s trustworthiness even further, regarding its *straightforwardness*.^11^ The strategy of (de-)legitimation applied is *reference by rationalization* as someone who is crazy does not act rational at all and therefore cannot be trusted to manage a pandemic accordingly. The lexeme *Wahn* is used ten times in the corpus, two times with explicit reference to Merkel (*Merkels Coronawahnsinn, Merkels #Coronawahnsinn, *tweets 21 and 18), one time with reference to the government and one time referring to Markus Söder, Minister-President of Bavaria (*Christian Social Union in Bavaria*, CSU) in another way, ›megalomania‹ (*#Söder s Größenwahn*), that is still affecting the politician’s trustworthiness (tweet 2). In another tweet it is claimed, that Söder ›is going crazy‹ (*durchdrehen*) (tweet 11), a verb that is also used with reference to Armin Laschet, Minister-President of the state of North Rhine-Westphalia (tweet 30). The supposed ›insanity‹ of the governing politicians is addressed furthermore by the terms *Irrsinn* (Eng. ›insanity‹), as in *Lockdown-Irrsinn* (tweet 24), and *Hysterie* (Eng. ›hysteria‹) used three times as a hashtag: *#Corona-Hysterie* (tweets 27, 29) and *#Corona-#Hysterie* (tweet 36).

The *topos of failure* is addressed by a broad variety of expressions ranging from objective ones like *Fehlentscheidungen *as in *folgenreichen Fehlentscheidungen der Bundesregierung *(Eng. ›consequential wrong decisions of the federal government‹), tweet 35, to colloquial ones used for personal attacks as in *#Merkel taugt nichts, von der Leyen*^12^*noch weniger* (Eng. ›#Merkel is no good, von der Leyen even less‹) (tweet 43). The extreme expression *Desaster* (Eng. ›disaster‹) is used four times to describe the government’s crisis management and its consequences for the *#Steuerzahler* (Eng. ›taxpayer‹) and *Bürger und Wirtschaft* (Eng. ›Citizens and economy‹), see tweets 3 and 19. In the following tweet AfD’s Federal spokesman Jörg Meuthen is referring to Merkel and Söder as agents of a ›disaster‹ leading to the ›Mass death of companies›:

(4) Prof. Dr. @Joerg_Meuthen betonte gegenüber der JF, **#Merkel und #Söder** scheine »in keiner Weise klar zu sein, was für ein **Desaster** sie derzeit anrichten«. Falls der #Lockdown bis Ostern verlängert werde, führe das zu einem **Massensterben von Unternehmen**. https://t.co/IzrwqpAeYS (tweet 9).

In the example (4) it is also alleged that Merkel and Söder are not aware of the consequences of their actions – a claim that tackles their *competence* even more. The (de-)legitimation strategy used by Meuthen is a *reference to a moral evaluation* that consists in capitalistic values of economic growth – a common argumentation scheme of the AfD.

The *performance* of Merkel, Spahn (CDU), Federal Minister of Health, and other ministers is attacked not only when it comes to their *competence* but also regarding *fairness* and *commitment* as the other two criteria of this component of trustworthiness. The following tweet combines all three criteria with the claim that Merkel and Spahn would ›stir up fears in an irresponsible way› (*fairness*), would lack of ›reasonable concepts and a plan B› (*competence*) and asking suggestively if ›the government does care about the people or only about gaining power for Spahn› (*commitment* – or *goodwill* in the Aristotelean terminology):

(5) RT @Tino_Chrupalla: Lockdown bis Weihnachten? **Spahn und Merkel schüren unverantwortlich Ängste**. **Vernünftige Konzepte? Plan B? Fehlanzeige**! Liegt der Regierung das **Volk am Herzen** oder **nur der Machtgewinn** für Spahn mit dem 3. Bevölkerungsschutzgesetz? https://t.co/hGSjTT4Dhp (tweet 22).

The claim that the crisis would be used for political reasons is repeated constantly by the AfD, also combined with a rather common allegation, that is, the government would spread chaos –another point of critique of its *performance* (see tweet 14). The lexeme *chaos* is used seven times in the tweets referring to the opening and closure of schools (*Schulchaos*) and the vaccination strategy (*Impf-Chaos*), also naming the government explicitly as the agent as in and *#GroKo-Impf-Chaos, Chaos-Regierung* and *Chaos-Verantwortliche #Jens Spahn* (tweets 33, 12, 16, 15). The federal and state governments’ supposed lack of *straightforwardness* is also addressed by the composition *#Corona-Wirrwarr* (Eng. ›confusion, mess›) in tweet 31.

The federal government is labeled ›the greatest danger of the #Corona crisis›, threating ›human live› and ›the future› (tweets 25, 13), ›destroying the economy and the society› (tweet 17) with its politics. The Minister-President of Bavaria, Markus Söder, is called a ›danger for the inner peace of our country› (tweet 11), while Chancellor Angela Merkel is ›becoming a risk› (tweet 39) and would ›ruin our country and our society› (tweet 39) – the verb *ruinieren* (Eng. ›to ruin›) is used four times, every time naming Merkel as the agent. The ›topos of threat› is addressed further by calling the government’s politics ›destructive› (tweet 20), claiming ›the Merkel government is placing the axe on our national economy› (tweet 37). As the examples show, the AfD uses the ›(de-)legitimation by reference to evaluation› excessively, considering ›economic values› decisive. Therefore, the personification of the economy is one rhetorical way to highlight its value as in a statement by Beatrix von Storch, Deputy Leader of the AfD, that is quoted in tweet 23, where it is claimed to be mortal: »We must protect human lives, but not let the economy die in the process!« (See also example 4 where the expression ›Mass death of companies› is used).

### Bolsonaro’s attacks on the trustworthiness of the media

In Bolsonaro’s corona discourse on Twitter (561 tweets and retweets on *@jairbolsonaro*) questions of trust, truth and disinformation are addressed directly. The term *verdade* (Eng. ›truth›) is used ten times, indicating that there is wrong information circulating that is being corrected by the Brazilian President and his ministers, according to the motto »A HORA DA VERDADE« (Eng. ›the hour of truth›)^13^ (tweet 13). The supposedly wrong information concerns the corona crisis in general, the vaccinations, the medication, the auxiliary program, and corruption involving public money for the crisis response (tweets 22, 2, 18, 13/19, 5). Whereas Bolsonaro announces to ›restore the truth about the vaccine in Brazil› and ›other truths› in his weekly live video (tweet 2) he blames the media indirectly to not telling the truth – as to say to lie and to spread wrong information –, noting:

(6) **Boa imprensa** não é a que mais critica ou elogia, mas **aquela que fala a verdade**. Teremos a vacina de forma segura e eficiente, para todos os voluntários, depois de certificada pela @anvisa_oficial (tweet 1).^14^

In another tweet Bolsonaro gets explicit, when stating that »C[c]ontrary to what the Brazilian media reported«

(7) […] a retirada do status de »uso emergencial hospitalar« pela FDA **na verdade** AMPLIA **o tratamento com hidroxicloroquina** nos EUA, permitindo o uso do medicamento, antes restrito, em qualquer ambiente, desde que receitado por um médico (tweet 18).^15^

While disregarding the health experts’ recommendations, Bolsonaro himself continuously gives advice on how to treat or avoid an infection with COVID-19 as can be seen in the example (7). During the first six month of the pandemic Bolsonaro recommends strongly the use of hydroxychloroquine, a medication used as a treatment for malaria and rheumatoid arthritis. As studies show, it does not bring any clinical benefit in patients with COVID-19 (cf. University of Oxford 2020) but can cause heart rhythm problems and serious side effects. The *Food and Drug Administration*, FDA (2020), »cautions against use of hydroxychloroquine or chloroquine for COVID-19 outside of the hospital setting or a clinical trial.« Bolsonaro continues to recommend its use anyways and dismisses the results as misrepresentation by the media:

(8) No mais, **essa mesma rede de TV desdenhou, debochou e desestimulou o uso da Hidroxicloroquina** que, mesmo não tendo ainda comprovação científica, salvou a minha vida e, como relatos, a de milhares de brasileiros (tweet 8).^16^

In the cited tweet (8) Bolsonaro refers to his own positive experiences with the use of hydroxychloroquine (*legitimation by personal authority*), exaggerating, it would have saved his life, and – limiting the certainty of his statement with a vague formulation – that of »thousands of Brazilians«. The observation that »even though it has no scientific proof yet« gives an indication of the little weight Bolsonaro gives to scientific findings, while referring to himself as a positive example is a common strategy in his rhetoric. In another tweet about the use of hydroxychloroquine in case of an infection Bolsonaro refers vaguely to »those who argue against Hydroxychloroquine, but don’t present any alternatives, I regret to inform you that I am very well with its use and, with the grace of God, I will live for a long time to come« (tweet 15). In one of the last statements on the subject on Twitter that dates to September 23, 2020, the president refers to the director of a hospital in Barretos, state of São Paulo, who »recommends Chloroquine for the treatment of Covid-19« (tweet 6). This authority (strategy of *legitimation by reference to expert authority*) begins his statement in the video that is linked by Bolsonaro as follows: »I’m not a doctor, but I’m the son of a doctor«. He then praises the »fantastic effect« of hydroxychloroquine. Bolsonaro considers the dismissal of the medicine as a matter of politization against him. He retweets a user’s thesis that »If Bolsonaro or Trump said that Chloroquine is bad for you, the media would support Chloroquine. If they said that oxygen is good for you, they would tell you to hold your breath« (tweet 23). Bolsonaro himself characterizes »most of the media‹ as hypocritical, claiming that ›Near the President the bloggers wear masks so they can attack him, but when they move away, they take them off« (tweet 30). In the attached video that is supposed to prove his point, three people take off their masks after leaving a crowd. Therefore, in this tweet a single case is used to legitimize a general assumption: That the media is lacking *straightforwardness*.

Bolsonaro uses Trump’s expression *fake news* on a regular basis, equating it with a lie: *Mais uma mentira (FAKE NEWS) *(Eng. ›Another lie‹)*.*^17^ On Twitter it appears twice in the corona discourse and four more times in other contexts. There is no explicit naming of the agent for this type of information when referring to interims Minister of Health, Eduardo Pazuello, a Brazilian Divisional general who is supposed to »clear some points« without »distortions or fake news« (tweet 14). The verb *esclarecer *(Eng. ›to clear‹) is used five times, not specificizing who is supposed to disseminate wrong information as shown in the following example:

(9) **Esclarecemos** que a referida MP, **ao contrário do que espalham**, resguarda ajuda possível para os empregados. Ao invés de serem demitidos, o governo entra com ajuda nos próximos 4 meses, até a volta normal das atividades do estabelecimento, sem que exista a demissão do empregado (tweet 27).^18^

Instead, Bolsonaro could not be more explicit on whom to blame for spreading wrong information, when urging his readers not to believe in the media:

(10)** NÃO ACREDITE NA MÍDIA FAKE NEWS**! SÃO ELES QUE PRECISAM DE VOCÊS! (tweet 29).^19^

Moreover, Bolsonaro uses the word *mentira* (Eng. ›lie‹) two times when referring to the media in the corona discourse, once with reference to »many in the press« as in »as mentiras de muitos da imprensa« (Eng. ›the lies of many of the press‹) (tweet 3) and in the second example being the one who is »disproving lies from the press« (»Desmentindo mentiras da imprensa«) (tweet 4). He also uses the verb *mentir* (Eng. ›to lie‹) one time, claiming that »a major part of the media« would lie »24 hours a day« (tweet 21).

The term *desinformação* (Eng. ›disinformation‹) that is crucial in the infodemic is used three times by Bolsonaro, always without naming explicitly an agent that is to be held responsible. The president claims that »disinformation has been a widely used weapon« in two tweets with a similar wording on two consecutive days:

(11) - Na guerra ao vírus **a desinformação foi uma arma largamente utilizada**.

- **O pânico foi disseminado** fazendo as pessoas acreditarem que só tinham um grave problema para enfrentar (tweet 10).^20^

(12) D‑ **A desinformação foi uma arma largamente utilizada**. **O pânico foi disseminado** fazendo as pessoas acreditarem que só tinham um grave problema para enfrentar.

- Não será fácil, mas havemos de recomeçar. BOM DIA A TODOS (tweet 11).^21^

As it is widely known that Bolsonaro alleges the media for »spreading panic« – another crucial predication concerning its trustworthiness regarding the criteria *fairness* and *transparency* – there is no need for an explicit reference. Additionally, it can be assumed that the effect for the creation of an overall climate of distrust might even be bigger when being vague about the agent. The analysis shows that Bolsonaro is using this kind of war rhetoric^22^ only a short period of time during the pandemic. This is somewhat plausible, as the Brazilian president downplays the threats of the pandemic. To portray the virus as an ›evil enemy‹ against whom you must go to war would be contradictory in this respect. In the third tweet in which the term ›disinformation‹ appears, Bolsonaro utilizes the rhetorical figure of a personification when claiming that it ›kills even more than the virus itself‹:

(13) A **desinformação mata mais até que o próprio vírus**. O tempo e a ciência nos mostrarão que **o uso político da Covid por essa TV trouxe-nos mortes que poderiam ter sido evitadas** (tweet 9).^23^

As »this TV network« is being named in the following sentence the interference to the one being blamed for disinforming can easily be made. Besides the allegation of disseminating wrong information the claim that the media would use the pandemic for political reasons is undermining its credibility even further. It is somewhat unusual that Bolsonaro is appreciating the science in this example (13), because usually he discredits advice given by health experts during the pandemic. He calls measurements ›extreme without planning and rationality‹ (tweet 26) and disregards them by not keeping distance himself, not wearing a mask and so on (cf. Leschzyk 2020). Bolsonaro claims that »the best cure« would be »to go back to work« (tweet 7), constantly referring to *economic values* to delegitimize the recommended actions. The president equates lives and jobs (tweet 16) and considers »the virus and unemployment« as »two problems that cannot be separated«, warning that »if the medicine is too much, the side effect will be much more disastrous« (tweet 24).

## Conclusion

It is striking that in the corona infodemic misinformation and even conspiracy theories are often promoted by popular political actors, regardless of whether they are running the government or in the opposition. In Germany, the opposition party AfD focuses on blaming the government, which they hold responsible for what they name *insane* and a *chaos*. They call the government *crazy* and *anti-democratic*, even evoking a *dictatorship*. In summary the answer to the question »Why shouldn’t we trust the government in the corona crisis?« given by the AfD is: The governing politics are lacking *competence* and *straightforwardness*. They have no *constitutional legitimation* and do not act according to our *economic values*.

The Brazilian president on the other hand is aiming at the media’s trustworthiness, also evoking an invisible actor with bad intentions as a scapegoat. He is attacking the media’s *credibility* focusing on the criterion *honesty*, accusing them to lie, to spread fake news and disinformation. His references and attributions can also be considered an attack on the media’s *performance* given the fact that the balanced information of the public is one of its main functions. Besides blaming the others Bolsonaro’s communication centers on himself: He makes alternative recommendations like using hydroxychloroquine, a medication that can cause severe side effects and brings no benefit in patients with corona disease. He often does not give any justifications for his claims and is creating a general climate of distrust by not naming agents explicitly.

Having in mind the ancient Aristotelean criteria of »How to appear a credible person« – *good sense, moral character*, and *goodwill* – it can be summarized that the AfD concentrates on the first one (as to say the government’s supposed lack of *expertise*), slightly touching *goodwill* when indicating that the governing politics do not care for the people, while Bolsonaro is focusing on the media’s supposedly inherently poor *character*. Both parties – the AfD in Germany and Jair Bolsonaro in Brazil – are sowing doubt and distrust constantly. This strategy is not only making people, institutions and the media seem unreliable, it also relieves the speakers from providing references of legitimation for their own claims as someone who reveals ›the truth‹ is supposed to tell ›the truth‹ himself.

In a pandemic it is crucial that scientifically proven recommendations reach the public. To do so, building trust in the institutions and in the media is key. For that reason, political communication should be clear, open, truthful, and up to date, as well as empathetic and consistent, so that trust can be gained, and measurements will be accepted. Nevertheless, at the end of the day, it is not enough to communicate that way – actions must be taken accordingly.

## Appendix

### Cited tweets

The following table contains tweets published via the accounts of *@AfD* and *@jairbolsonaro*. These can be viewed in full via the attached links, inserting https://twi2tter.com/AfD/status/ respectively https://twitter.com/jairbolsonaro/status/ beforehand (accessed on 11.03.2021). The highlighting in bold is mine. The listing is in reverse chronological order.

**Table 4:** Cited tweets AfD

**Nr.**	**Date**	**Embedding**	**Post**	**Tweet-ID**
1	14.02.2021	photo	RT @StBrandner: #5Fragen5Antworten […]: **#Coronakratur** bis in alle #Ewigkeit? Wird es jemals ein #Lockdownende geben? **Wieso tanzen alle #Ministerpräsidenten nach #Merkel|s Pfeife?** Dies und noch viel mehr in der aktuellen Folge. […] https://t.co/mN7n7nhoWp #5f5a #AfD #Berlin #Bundestag #Brandner https://t.co/qQ3y6qJF0k	1360910064698200068
2	09.02.2021	link	Frust pur: Sogar #CSU-Abgeordnete vergleichen **#Söder s Größenwahn** mit Caesar! Zeichen der Spaltung? Sogar CSU-Abgeordneten sind völlig frustriert über radikale #Corona-Politik! »Wir brauchen mehr #Landesvater und weniger #Caesar!« https://t.co/xDdzu4zn7n	1359072809918545921
3	03.02.2021	image w\ text	»Im Großen und Ganzen nichts schief gelaufen [sic]« Mit diesen weltfremden Worten verharmlost **#Merkel ihr #Impf-Versagen**. Nicht nur, dass es unter ihrer Führung im Schneckentempo vorangeht – ihr Impf-Management ist auch **für den #Steuerzahler ein Desaster**. #afd https://t.co/NzLSHZrPWThttps://t.co/JJmkIIpYlw	1357018542894374914
4	02.02.2021	image w\ text	**Sollte** »#Lockdown« nicht bereits am 31.01.21 enden? **Wurde nicht suggeriert**, dass die Verlängerung keineswegs beschlossene Sache & Debatte keine **Scheindebatte** wäre? #Merkel s Aussage ist **Verhöhnung** – **für die Bürger, unser Land, unsere Verfassung**! #afd https://t.co/sCgUA7yG2Mhttps://t.co/7gcKmRgIIH	1356656486185914370
5	27.01.2021	image w\ text	#Merkel wirkt zunehmend dünnhäutig bei aufkommender Kritik. Zuletzt beklagte sie sich beim Kanadischen Premierminister darüber, dass **sie wegen des Impf-Versagens** »jeden Tag von den deutschen Medien kritisiert wird.« Kritik ungleich Majestätsbeleidigung: https://t.co/7eCwt89AvMhttps://t.co/EIN5yZL2WR	1354481857182769157
6	26.01.2021	image w\ text	**#Politik-Versagen**: Über 170.000 Unternehmen vor der #Insolvenz! Zweifellos werden die #Altparteien auch weiterhin versuchen, den kommenden #Wirtschaft s‑GAU ausschließlich auf das #Corona-Virus zurückzuführen. Eine #Konjunktur-Umfrage des #DIHK zeigt: https://t.co/yVDXAK71l3https://t.co/FyvLWElqC0	1354119457938010112
7	24.01.2021	image w\ text	++ #Corona-Schulausfall kostet zukünftigen Generationen bis zu 3,3 Bio. Euro! ++ Es sind erschreckende Zahlen: Laut einer Berechnung des #ifo-Instituts kostet zukünftigen Generationen 3.300 Mrd. €. **#Regierungsversagen** STOPPEN! https://t.co/Ti1s7QOtVthttps://t.co/NsoZOlOp68	1353364453925003264
8	13.01.2021	video	RT @AfDimBundestag: +++Wir lehnen jede #Impfpflicht ab!+++ Sebastian @S_Muenzenmaier, stellv. Vorsitzender der #AfD-Fraktion, rechnet in seiner Rede mit der fatalen #Corona-Politik von Jens #Spahn und Angela #Merkel ab: »**Verrückt gewordene Politiker spielen sich als Corona-Sheriffs auf**!« https://t.co/2XeBuCEIOq	1349611980764667904
9	12.01.2021	photo & link	Prof. Dr. @Joerg_Meuthen betonte gegenüber der JF, **#Merkel und #Söder** scheine »in keiner Weise klar zu sein, was für ein **Desaster** sie derzeit anrichten«. Falls der #Lockdown bis Ostern verlängert werde, führe das zu einem **Massensterben von Unternehmen**. https://t.co/IzrwqpAeYS	1349001905809326080
10	11.01.2021	link	Thüringen: **Versagen der Landesregierung** verursacht Einzelhandelssterben - https://t.co/fK4PEaaqFK	1348651533290897414
11	11.01.2021	photo & link	RT @Joerg_Meuthen: Guten Morgen de! **Söder dreht durch**: Er vergleicht Corona mit der Pest und spricht von der Gefahr einer »Corona-RAF« - und das auch noch in direkter Verbindung mit unserer Bürgerpartei. Dieser Mann ist eine **Gefahr für den inneren Frieden unseres Landes**. ➔ https://t.co/1nqy2Sn9L5https://t.co/fgFYSdwdEO	1348541766237114369
12	11.01.2021	photo & link	Lauterbach widerspricht Merkel: **Impf-Chaos geht immer weiter**! - https://t.co/BP8nOALHrm	1348636219417251843
13	08.01.2021		RT @Tino_Chrupalla: Unfassbar! Harter #Lockdown in #Sachsen bis 7. Februar verlängert. Geschäfte geschlossen. Keine Kinderbetreuung. Schulbildung digital. Winterferien gestrichen. **Zukunft des Landes gefährdet**! Wie wollen Sie das der zukünftigen Generation gegenüber rechtfertigen, #MPKretschmer?	1347655010373562371
14	06.01.2021	image w\ text	#Merkel entmachtet #Spahn: **#GroKo-Impf-Chaos eskaliert**! Inmitten der #Corona-Krise, um deren Lösung sich die Altparteien **angeblich bemühen**, dominieren Befindlichkeiten und **Profilierungszirkus** das Bild. Diese #Regierung muss krachend abgewählt werden! https://t.co/5aBlFbFFIphttps://t.co/13RK8MjCuX	1346849280561188865
15	06.01.2021		RT @Tino_Chrupalla: **#Impfstoff -Desaster** führt zu offenem Machtkampf in #BuReg. Es wird berichtet: »#Merkel bremst Impfstoff-Kauf aus«. Wer beschuldigt die Kanzlerin? Der **Chaos-Verantwortliche #Jens Spahn**? Für seine Karriere? #COVID19 offenbart die Niedertracht und Unfähigkeit dieser Regierung.	1346061589095587840
16	30.12.2020		RT @Tino Chrupalla: **Pleiten‑, Pech- und Pannen-Minister Jens #Spahn**: Erst fehlten #Masken, dann **floppte** die #CoronaWarnApp jetzt gibt es keinen #COVID19 - Impfstoff. Wann räumt diese **Chaos-Regierung** endlich ihr **Scheitern** bei #COVID19 ein?«	1344567545739808769
17	13.12.2020	image w\ text	RT @StBrandner: »Statt Risikogruppen wirksam zu schützen, wie wir es der Regierung seit Monaten vorbeten, **zerstört man Wirtschaft und Gesellschaft**: diese **Politik ist gefährlich für unser Land**.« Ganze PM hier: https://t.co/HDtI4v33IF #AfD #Berlin #Bundestag #Brandner https://t.co/0XdoW7kINm	1338489138652319745
18	13.12.2020	photo	RT @StBrandner: **Merkels #Coronawahnsinn **[…]: #Klatschen gegen Kälte, #Radfahren gegen #ÖPNV und Pullover vor dem 27.12. kaufen. Tolle […] **Tipps unserer Regierung und anderer #Wahnsinn**! […] https://t.co/diFKopALGD #5Fragen5Antworten #5f5a #Berlin #Bundestag #Brandner https://t.co/7yRN1zyq1Z	1338075000700592134
19	13.12.2020	image w\ text	RT @Alice_Weidel: Dieser #Lockdown-Beschluss ist ein Offenbarungseid für die politisch Verantwortlichen und ein **Desaster für Bürger und Wirtschaft**. Nötig ist ein stringentes Schutzkonzept für gefährdete Bevölkerungsgruppen. Die Holzhammer-Methode »Lockdown« ist dagegen kontraproduktiv! #AfD https://t.co/j8pQSr0er6	1338074869787922434
20	09.12.2020	video	In der heutigen #Generaldebatte rechnet @Alice_Weidel gnadenlos mit der **zerstörerischen Politik von Angela #Merkel** ab. […] https://t.co/T8qNIRusdP	1336621825489596416
21	24.11.2020	link	RT @StBrandner: […] Stephan Brandner: **Merkels Coronawahnsinn geht weiter**: kein **Einsperren **über Weihnachten! https://t.co/w6Ay6DwZKj	1331209753310203905
22	12.11.2020	photo & link	RT @Tino_Chrupalla: Lockdown bis Weihnachten? **Spahn und Merkel schüren unverantwortlich Ängste**. **Vernünftige Konzepte? Plan B? Fehlanzeige!** Liegt der Regierung das **Volk am Herzen oder nur der Machtgewinn für Spahn **mit dem 3. Bevölkerungsschutzgesetz? https://t.co/hGSjTT4Dhp	1327151021555118080
23	06.11.2020	video	RT @AfDimBundestag: +++**#Merkel-#Lockdown ruiniert unser Land!**+++ @Beatrix_vStorch kritisiert in der Debatte zu den Verschärfungen der #Corona-Maßnahmen die Unverhältnis-mä-ßig-keit: »Wir müssen Menschenleben schützen, dürfen dabei aber **die Wirtschaft nicht sterben lassen**!« #AfD https://t.co/TVHCgBhnQz	1324687055805362179
24	05.11.2020	video	+++**Lockdown-Irrsinn**: Die **Medizin darf nicht schlimmer sein als die Krankheit**!+++ @GottfriedCurio fordert im Namen der #AfD-Fraktion die **verfassungswidrige** Umgehung des Parlaments bei Corona-Maßnahmen zu beenden. Die Beschlüsse des #Corona-Gipfels müssen rückgängig gemacht werden.	1324371528381857795
25	02.11.2020	photo & link	RT @Tino_Chrupalla: **#BuReg ist größte Gefahr der #Corona -Krise**: Pflegenotstand auf Intensivstationen **gefährdet Menschenleben**. Das ist die Politik von **#MerkelMussWeg und #Spahn**. https://t.co/uvVBFAQgKg	1323224934013931520
26	01.11.2020	photo	RT @StBrandner: Meine Rede: Die **millionenfache Verfassungsbrecherin #Merkel** und ihre Helfershelfer einsperren! https://t.co/f2qfTRCxbx #Berlin #Bundestag #Brandner https://t.co/1P4jzuD7c5	1322815455493525505
27	01.11.2020	image w\ text	[…] #Merkel und Co. ruinieren mit **#Corona-Hysterie** die Wirtschaft und machen das gesellschaftliche Klima kaputt. Reaktion:[…] #Hausverbot für Merkel + Minister in Kneipen, Restaurants und Hotels. Richtig so! Hier bekommt Merkel bereits jetzt kein Bier mehr: https://t.co/0nb8bWDn1whttps://t.co/CgqwcvbRM4	1322821187446181888
28	29.10.2020	video	RT @AfDimBundestag: Wir werden von einem Kriegskabinett regiert! Alexander #Gauland, Vorsitzender der #AfD-Fraktion, antwortet auf die #Regierungserklärung von Angela #Merkel: »Eine **#Corona-Diktatur auf Widerruf** verträgt sich nicht mit unserer freiheitlich demokratischen Grundordnung!« #Lockdown2 https://t.co/yOPtMJKWqV	1321744656175124480
29	17.10.2020	image w\ text	Ständiges Lüften: #Bildungspolitik in **#Corona-Hysterie** treibt immer wildere Stilblüten! Im #Winter sollen »Pullover, Schals und Decken zur Grundausstattung der Schülerinnen & Schüler gehören!« Mehr erfahren: https://t.co/GNjfRYbXsqhttps://t.co/ljjNBxkq0J	1317480805510336516
30	16.10.2020	image w\ text	RT @Tino_Chrupalla: Da haben wir ja nochmal Glück gehabt. **Der tägliche #Corona Wahnsinn**! Der nächste der **durchdreht**. Der **»neue« Kanzler #Laschet** […]**#Panikorchester** https://t.co/UWnFqe5Llo	1317162888969191424
31	09.10.2020	photo & link	RT @Tino_Chrupalla: Heute im Morgenmagazin ging es um das **#Corona-Wirrwarr der Bundes- und Landesregieren**. Schalten Sie ein! […] https://t.co/10yxsSBM4J	
32	30.09.2020	video	RT @Alice_Weidel: **Merkel macht Rechtsbruch zum politischen Prinzip**! Die #GroKo glänzt durch **Totalversagen**. Von Wirtschaft über Klima und Justiz bis hin zu Flüchtlings-politik und **#Corona-Wahn** – alles haben #Merkel und ihre Regierung **in den Sand gesetzt**. Meine **Generalabrechnung** seht Ihr im Video: https://t.co/CKTKcvgd4W	1311214389748432896
33	08.08.2020	image w\ text	RT @Joerg_Meuthen: Guten Morgen de! Das **Schulchaos **der letzten Monate darf keinesfalls fortgesetzt werden. Die Schulen sind konsequent im **Normalbetrieb** zu öffnen, Ausnahmen darf es nur bei echten Corona-Hotspots geben, und dies auch dort nur in engster zeitlicher Begrenzung. https://t.co/AtWMVPzR7Vhttps://t.co/5mOBuji072	1292513047723081732
34	02.07.2020	image w\ text	RT @Joerg_Meuthen: Guten Morgen de! Der Corona-Lockdown hat gezeigt: Verkehrsaufkommen & Stickoxid-Werte hängen kaum zusammen. Merkel & Co. weigern sich trotzdem, die Fahrverbote zurückzunehmen. Das ist **faktenbefreit & ideologisch verbohrt**, und zwar zum Nachteil der Bürger! https://t.co/VoX2gka8JFhttps://t.co/W53FpvHB7f	1278626437252161536
35	30.05.2020	link	RT @AfDimBundestag: Wir als #AfD-Fraktion werden einen **Untersuchungsausschuss zum #Corona-Krisenmanagement der Regierung** beantragen! Die **folgenreichen Fehlentscheidungen der Bundesregierung müssen aufgearbeitet werden**. #Covid_19 #Bundestag #Merkel https://t.co/pqbJVR4m7G	1266704976337932288
36	13.05.2020	image w\ text	Die Proteste gegen Beschneidung und teilweise Abschaffung der #Grundrechte in der **#Corona-#Hysterie** werden immer größer. Zehntausende gehen auf die Straße und fordern ein Ende des »#Lockdowns«. Mainstream-Medien diskreditieren diese #Demonstranten ... https://t.co/3mGcw84Em7https://t.co/J9wBmnHgUR	1260537537048829953
37	30.04.20202	image w\ text	RT @Tino_Chrupalla: Die **Merkel-Regierung legt die Axt an unsere Volkswirtschaft**: 10 Mio. #Kurzarbeiter, 2,6 Mio. Arbeitslose, drohende 50.000 Insolvenzen im Einzelhandel, fast jeder zweite Gastronomiebetrieb pleite. Deutschland rutscht in die schwerste Rezession der Nachkriegszeit. #Merkel muss weg! https://t.co/Uk45CJc77f	1255836113501126658
38	29.04.2020		RT @Tino_Chrupalla: **Bittere Wahrheit** für #Pflegekräfte: Das Lob der Bundesregierung für ihre Leistung in der #Coronakrise war nur ein **Lippenbekenntnis**. **Statt der versprochenen** 1500 Euro Bonus bekommen sie nur 1000 Euro. **Merkel Spahn sollten sich schämen**!	1255598988851625991
39	21.04.2020	image w\ text	RT @Tino_Chrupalla: Die Deutschen verzichten derzeit auf viel, um andere nicht zu gefährden. Mit ihrem **überheblichen Eingreifen** in die Bürgerrechte lässt Angela Merkel die Stimmung kippen. Im Kampf gegen das #Coronavirus **wird sie zum Risiko**. https://t.co/vCjxyJIUo0	1252589324610273281
40	21.04.2020	video	RT @Beatrix_vStorch: #Merkel erklärt #lockdown - Exit-Debatte zu #Öffnungsdiskussionsorgie. Was für eine **demokratieverachtende Haltung**. Hunderttausende Bürger kämpfen um ihre schiere Existenz und die Kanzlerin erklärt deren Fragen zu einer »Orgie«. Was für eine **#Entgleisung**. #COVIDー19 https://t.co/xxMeinyHKY	1252503204543238144
41	20.04.2020	image w\ text	RT @StBrandner: »#Öffnungsdiskussionsorgien«: **Merkel ruiniert unser Land und unsere Gesellschaft** – Öffnungsdiskussion zwingend erforderlich! Ganze Mitteilung hier: https://t.co/Vsu6gW3nMY »**Sie ruinieren unser Land**!« #AfD #nurnochAfD #Berlin #Bundestag #Brandner https://t.co/ZTMP3nA6BG	1252194029405704192
42	18.03.2020		**»Die Kanzlerin führt nicht, sie reagiert wieder nur** und sie sagt nicht, wie es weitergeht« @Beatrix_vStorch im #ZDFspezial zur #Regierungserklärung von #Merkel zu #CoronaVirusDE.	1240351054979571712
43	02.02.2020		RT @StBrandner: Läuft irgendwie überhaupt nicht so, wie von #Altparteien, #Staatsfunk & #Einheitsmedien gewünscht und rumtrompetet: #Brexit klappt, #Trump bleibt im Amt, #Coronavirus ist in Deutschland und gefährlich, **#Merkel taugt nichts, von der Leyen noch weniger**... usw.	1224063836887666700

**Table 5:** Cited tweets Jair Bolsonaro

**Nr.**	**Date**	**Embedding**	**Post**	**Tweet-ID**
1	12.01.2021	video	- **Boa imprensa** não é a que mais critica ou elogia, mas **aquela que fala a verdade**. - Teremos a vacina de forma segura e eficiente, para todos os voluntários, depois de certificada pela @anvisa_oficial - Assista é só depois comente. . Link no YouTube: https://t.co/C5f1LaKvc9	1348936168050749442
2	07.01.2021	video	- **O restabelecimento da verdade sobre a vacina no Brasil**. @minsaude - Na live das 19h mais verdades. - Bom dia a todos. Link no YouTube: https://t.co/zmSB0HsQkB	1347151224630599680
3	10.12.2020		- Temas da semana: . Zerados impostos no combate ao covid e outros; @MinEconomia . Brasileiro preso na Rússia; . Vacina; @ItamaratyGovBr @ernestofaraujo . Economia com reflexos positivos e derivações na pandemia; . @MTurismo e **as mentiras de muitos da imprensa**;	1337165812101156864
4	03.12.2020		[…] Temas: . Trabalho da @policiafederal; . Investimentos em obras; . Crescimento do turismo; . Energia solar, nuclear e abastecimento; . Ações do @mmeioambiente e @Minas_Energia; . Grafeno e nióbio; . Crescimento da economia; . **Desmentindo mentiras da imprensa**; . Covid-19.	1334624622826893314
5	09.10.2020		- AGENDA E TEMAS: PARTE 4 . Novo Programa de Mineração e Desenvolvimento com preservação ao meio ambiente; . Respeito ao limite do teto de gastos; . **A verdade sobre** a lava-lato, que continua, »**covidão«**, operações sobre irregularidades na Petrobras e outros;	1314367931367686144
6	23.09.2020	video	- Henrique Prata, **diretor do Hospital do Amor de Barretos, recomenda a Cloroquina para o tratamento da Covid-19**. No YouTube: https://youtu.be/KpIYo3Xg1P8	1308572054019543040
7	11.08.2020	video	- No Brasil são 38 milhões de informais. - **Volta ao trabalho, o melhor remédio**. Nosso canal no YouTube: https://youtu.be/_PuQDKNU37A	1292974736625020929
8	09.08.2020		- No mais, **essa mesma rede de TV desdenhou, debochou e desestimulou o uso da Hidroxicloroquina** que, mesmo não tendo ainda comprovação científica, **salvou a minha vida** e, **como relatos, a de milhares de brasileiros**.	1292523257288167425
9	09.08.2020		- **A desinformação mata mais até que o próprio vírus**. O tempo e a ciência nos mostrarão que **o uso político da Covid por essa TV trouxe-nos mortes** que poderiam ter sido evitadas.	1292523327328747522
10	13.07.2020	video	- **Na guerra ao vírus a desinformação foi uma arma largamente utilizada**. - **O pânico foi disseminado** fazendo as pessoas acreditarem que só tinham um grave problema para enfrentar. - Por @GFiuza_Oficial:	1282710338585100289
11	12.07.2020		D- **A desinformação foi uma arma largamente utilizada**. **O pânico foi disseminado** fazendo as pessoas acreditarem que só tinham um grave problema para enfrentar. - Não será fácil, mas havemos de recomeçar. BOM DIA A TODOS.	1282319055274283008
12	12.07.2020		C- A realidade do futuro de cada família brasileira deve ser **despolitizada da pandemia**. Os números reais **dessa guerra** brevemente aparecerão.	1282319015302529025
13	12.07.2020		A- **A HORA DA VERDADE**: - A situação só não está pior pelas ações do @govbr que foi ao socorro das pequenas e médias empresas, arranjou recursos para estados e municípios e está pagando **Auxílio Emergencial** de R$ 600,00 para mais de 60 milhões de pessoas.	1282318595696070656
14	09.07.2020	video	**Sem distorções ou fake news**, @minsaude Eduardo Pazuello **esclarece pontos** sobre sua gestão.	1270440778268098561
15	08.07.2020	photo	D- **Aos que torcem contra a Hidroxicloroquina**, mas não apresentam alternativas, **lamento informar que estou muito** bem com seu uso e, com a graça de Deus, viverei ainda por muito tempo.	1280849256593702918
16	08.07.2020		C- Nenhum país do mundo fez como o Brasil. **Preservamos vidas e empregos** sem propagar o pânico, que também leva a depressão e mortes. Sempre disse que **o combate ao vírus não poderia ter um efeito colateral pior que o próprio vírus**.	1280849187580633094
17	18.06.2020		C2. Assim se mede a carga viral no início do protocolo e ao longo do tratamento, que dura 5 dias e mais 9 dias de observação. **Um outra arma poderosíssima** contra os efeitos da Covid-19.	1273553863732330496
18	16.06.2020	video	- **Ao contrário do que divulgou a mídia brasileira**, a retirada do status de »uso emergencial hospitalar« pela FDA **na verdade** AMPLIA **o tratamento com hidroxicloroquina** nos EUA, permitindo o uso do medicamento, antes restrito, em qualquer ambiente, desde que receitado por um médico.	1272985282904801286
19	14.06.2020	video	i1- **A verdade sobre a grande operação do auxílio emergencial**. @MinCidadania @onyxlorenzoni http://youtu.be/w9JhPpQ4NIM i2- @govbr recupera quase R$ 30 milhões em fraudes e erros do auxílio emergencial. Operação em andamento. https://bit.ly/3hrrImo	1272175358918496256
20	13.05.2020	picture	DIA DO ENFERMEIRO - **Na atual guerra que enfrentamos**, existem aqueles que **estão lutando na linha de frente** pelas nossas vidas. - Nesta data, parabenizamos todos os enfermeiros do Brasil pelo seu dia.	1260369368191578114
21	11.05.2020	video	- Hidroxicloroquina e, mais uma vez, **grande parte da mídia é desmascarada** sobre o uso do cartão corporativo. **Lixo**! **Mentem 24 horas ao dia**!	1259820202117775360
22	10.05.2020	link	- **A verdade**: covid-19.	1259301707873497091
23	13.04.2020		RT @jeffs_araujo35: Se Bolsonaro ou Trump dissesse que a Cloroquina faz mal, **a mídia apoiaria a Cloroquina**. Se dissessem que o oxigênio faz bem, mandariam prender a respiração. Paporra.	1249504564098879489
24	30.03.2020	link	Temos **2 problemas que não podem ser dissociados: o vírus e o desemprego**. Ambos devem ser tratados com responsabilidade. Mas **se o remédio for demasiado o efeito colateral será muito mais desastroso**.	1244576384271503360
25	26.03.2020		- É mais fácil **fazer demagogia** diante de uma **população assustada**, do que **falar a verdade**. Isso custa popularidade. Não estou preocupado com isso! **Aproveitar-se do medo das pessoas para fazer politicagem** num momento como esse é coisa de COVARDE! **A demagogia acelera o caos**.	1242983915649994755
26	24.03.2020		- A epidemia afeta diretamente a todos, mas **medidas extremas sem planejamento e racionalidade** podem ser ainda mais nocivas do que a própria doença no longo prazo. Quando falamos em proteger em-pregos, também estamos falando de preservar a vida das pessoas. É isso que faremos!	1242261759878201344
27	23.03.2020		**Esclarecemos** que a referida MP, **ao contrário do que espalham**, resguarda ajuda possível para os empregados. Ao invés de serem demitidos, o governo entra com ajuda nos próximos 4 meses, até a volta normal das atividades do estabelecimento, sem que exista a demissão do empregado.	1242071606274535426
28	23.03.2020		- Nossas Forças Armadas, sempre lembradas em tempos difíceis, estão à disposição p/ dar todo apoio possível aos Estados e Municípios do país **na guerra contra o coronavírus**, com logística, transporte de profissionais de saúde e materiais, postos de triagem, etc. **JUNTOS VENCEREMOS**!	1241879151214288896
29	13.03.2020		- **NÃO ACREDITE NA MÍDIA FAKE NEWS**! - SÃO ELES QUE PRECISAM DE VOCÊS!	1238491455200649219
30	15.02.2020	video	- Perto do Presidente os blogueiros usam máscara para poderem atacá-lo, mas quando se afastam as tiram. - **Essa é a hipocrisia de grande parte da mídia**. - São Francisco do Sul/SC. Link no YouTube: https://youtu.be/hWO9oR6LTgk	1361416071316561921

## Footnotes

1 For characteristics on conspiracy theories see Michael Butter (2018).

2 Eng. ›With little advertising time on television, Bolsonaro, following D. Trump’s example, is betting on Tweets, Facebook and WhatsApp posts, thus centralizing his communication channel with voters in the digital media. This and all other translations are by the author and are as literal as possible.‹.

3 A more detailed typologization of misinformation can be found in »Types, sources, and claims of COVID-19 misinformation« by Brennen et al. (2020).

4 I have applied and described the concept of trustworthiness by Renn (2019) more in detail in my article »Diskreditierung in der Krise. Rhetorische Angriffe auf die Vertrauenswürdigkeit der österreichischen Regierung« (in print).

5 Van Leeuwen has described the categories for the first time in the unpublished manuscript »The Grammar of Legitimation« (School of Media, London College of Printing). They are presented in van Leeuwen/Wodak (1999, pp. 104 f.) and van Leeuwen (2013) among others.

6 See tweets 39, 34, 34 and 42.

7 All translations of AfD’s tweets are mine. The complete German wording can be found in the appendix (table 4), followed by the corresponding Twitter-ID.

8 The *Große Koalition* is a coalition of the parties CDU/CSU and SPD (*Social Democratic Party of Germany*).

9 As it is common in right wing populism, the AfD positions itself as anti-establishment, calling the established parties ›old parties‹ in a derogatory way.

10 See tweets 7, 3, 5, 6 and 10 in the appendix.

11 Even more explicit in a tweeted quote of Sebastian Münzenmaier, member of the Bundestag: *Verrückt gewordene Politiker spielen sich als Corona-Sheriffs auf!* (Eng. ›Politicians gone mad act as Corona sheriffs‹, tweet 8).

12 Ursula von der Leyen (CDU) is the President of the European Commission (2019-).

13 All translations of Bolsonaros’s tweets are mine. The complete Portuguese wording can be found in the appendix (table 5), followed by the corresponding Twitter-ID.

14 Eng. ›Good press is not the one that criticizes or praises the most, but the one that tells the truth. We will have the vaccine, safe and efficient, for all volunteers, after certification by @anvisa_oficial.‹.

15 Eng. ›[...] the removal of the status ‘hospital emergency use’ by the FDA actually EXTENDS hydroxychloroquine treatment in the US by allowing the previously restricted use of the drug in any setting, as long as it is prescribed by a doctor.‹.

16 Eng. ›Moreover, this same TV network disdained, debauched and discouraged the use of hydroxychloroquine that, even without scientific proof, saved my life and, as reported, that of thousands of Brazilians.‹.

17 This tweet is not part of the corona discourse, it can be accessed through https://twitter.com/jairbolsonaro/status/1314290803712368640 (22.06.2021).

18 Eng. ›We clarify that the mentioned MP, contrary to what is being spread, provides potential support for the employees. Instead of being fired, the government will help them for the next 4 months, until the normal activities of the establishment are resumed, without dismissal of the employee.‹.

19 Eng. ›DON’T BELIEVE THE FAKE NEWS MEDIA! IT IS THEM THAT NEED YOU!‹.

20 Eng. ›‑ In the war on the virus disinformation was a widely used weapon. - Panic was spread by making people believe that they only had one serious problem to face.‹.

21 Eng. D‑ Misinformation was a widely used weapon. Panic was spread by making people believe that they only had one serious problem to face. - It won’t be easy, but we will start over. GOOD MORNING TO ALL.

22 The word *weapon* (Pt. *arma*) is used three times (tweets 10, 11, 17), the word *war* (Pt. *guerra*) four times (tweet 10, 12, 20, 28), for example, as in the following tweet 20: »DIA DO ENFERMEIRO - **Na atual guerra que enfrentamos**, existem aqueles que **estão lutando na linha de frente pelas nossas vidas**. - Nesta data, parabenizamos todos os enfermeiros do Brasil pelo seu dia.« (Eng. ›NURSES‹ DAY - In the current war we are facing, there are those who are fighting in the frontline for our lives. - On this date, we congratulate all the nurses in Brazil for their day.‹).

23 Eng. ›Disinformation is more deadly than the virus itself. Time and science will show us that this TV’s political use of Covid has brought us deaths that could have been avoided.‹.
